# Microplastic Pollution and Its Ecological Risks in the Xisha Islands, South China Sea

**DOI:** 10.3390/toxics13030205

**Published:** 2025-03-12

**Authors:** Wenchao Wei, Yun Zhang, Licheng Wang, Qiao Xing, Jun Xiang, Yuquan Zhang, Qifei Peng, Yongfu Chen, Yufeng Hu, Yini Ma, Ling Mo

**Affiliations:** 1 Hainan Research Academy of Environmental Sciences, Haikou 571127, China; weiwenchao0425@163.com (W.W.); wanglch388@sohu.com (L.W.); xingqiaohn@126.com (Q.X.); xiangjungd2020@163.com (J.X.); zhangyuquan2217@163.com (Y.Z.); b546770@163.com (Q.P.); chen_n0820@163.com (Y.C.); hyf1035819535@163.com (Y.H.); 2 College of Resources & Environment of Huazhong Agricultural University, Wuhan 430070, China; 3 Hainan Medical University—The University of Hong Kong Joint Laboratory of Tropical Infectious Diseases, Key Laboratory of Tropical Translational Medicine of Ministry of Education, School of Basic Medicine and Life Sciences, Hainan Medical University, Haikou 571199, China; yunzhang_hnmu@163.com; 4 College of Life and Environmental Science, Central South University of Forestry and Technology, Changsha 410004, China; 5 School of Environmental Science and Engineering, Hainan University, Haikou 570228, China

**Keywords:** microplastic, characterization of microplastic pollution, ecological risk assessment

## Abstract

China is facing increasing marine microplastic pollution. Despite the fact that the South China Sea is the largest marine area in China, the ecological danger and present state of microplastic contamination in this region have not been systematically and comprehensively investigated. This study analyzed the abundance, distribution, and characteristics of microplastics in different environmental media and biological samples from the Xisha Islands in the South China Sea, and then the ecological risk assessment of microplastic pollution in this area was conducted. The findings indicated that the quantities of sediments, soil, water, fish, and birds were 41.56 ± 19.12 items/kg, 92.94 ± 111.05 items/kg, 2.89 ± 1.92 items/L, 2.57 ± 2.12 items/ind, and 1.702 ± 1.50 items/ind, respectively. By evaluating the pollution load index (PLI), polymer hazard index (PHI), and potential ecological risk index (PERI), the PLI of the Xisha Islands in the South China Sea as a whole indicated that the hazard level was slightly polluted, the PHI was at a high-risk level, and the PERI samples were at no risk, except for the soil and seawater, which were at a medium-risk level.

## 1. Introduction

Microplastics, characterized as plastic particles of less than 5 mm, have received much attention recently due to their pervasive prevalence throughout several ecosystems [1]. Many places, from surface seawater to deep-sea sediments and from subtropical ocean circulations to polar and freshwater systems, as well as in tributaries, have been found to be contaminated by microplastics [2,3]. Upon ingestion by organisms, microplastics can obstruct their tissues and organs [4], thus impacting their energy stores and resulting in neurotoxicity, behavioral anomalies, stunted growth, diminished reproductive potential, and, finally, mortality [5]. Ingested microplastics can build up in the human body from seafood eating, and microplastics in the human placenta have recently been reported to have serious effects on fetal development [6].

The South China Sea is a large semi-enclosed marginal sea that has an average depth of approximately 1200 m and covers an area of almost 3.5 million square kilometers [7]. The Xisha Islands (15°47′~17°08′ N, 110°10′~112°55′ E), situated in the northwestern region of the South China Sea, are composed of the Yongle and Xuande Islands [8], which have extremely rich marine biological resources and high biodiversity [9]. The South China Sea ranks among the most significant and representative habitats for birds in the Western Pacific Ocean, with as many as 60 species of birds [8]. Island ecosystems are unique and fragile ecosystems that are highly susceptible to the influence of the surrounding waterbodies and to natural and human disturbances, and, because of the limited size of many islands, they are prone damage and face serious ecological and environmental problems. In addition, the South China Sea is adjacent to China and a number of Southeast Asian countries, and the pollution and ecotoxicity of the South China Sea waters by pollutants from the industrial and agricultural activities of these surrounding countries is complex, and is one of the threats to the ecological diversity of the islands in the South China Sea [10].

Studies in recent decades have shown that microplastics are widely present in marine environmental media (air, water, soil, sediment) and organisms (fish, benthic animals) in different areas. Microplastics were detected in sediments (72.0–171.8 items/kg), soil (740.1 items/kg), seawater (0.06–7.79 items/m^3^), and fish (1.0 ± 1.2 items/ind) in the Bohai Sea [11,12]. In addition, microplastics were also detected in sediments in the Black Sea (106.7 items/kg), Yellow Sea (560–4205 items/kg) [13,14], and seawater from the Western Pacific Ocean (240 items/m^3^) and the East China Sea (0.14 ± 0.12 items/m^3^) [15,16]. The main sources of microplastics in the Black Sea and Yellow Sea may be river runoff, mariculture, garbage eddies, and anthropogenic activities [12,13,14].

Compared with other areas, anthropogenic activities and fish farming in the South China Sea are rare, so the microplastic contamination in environmental media and organisms in the South China Sea has received less attention. However, under the influence of typhoons and the southwest monsoon [17], the characteristics of microplastic contamination in the South China Sea should not be neglected. Although previous studies have provided good background information on the abundance of microplastics in the organisms of the South China Sea islands, our understanding of the pollution status of microplastics in the organisms of the South China Sea islands is still limited. With the in-depth research on microplastic pollution, more and more studies have been conducted to assess the ecological risk of microplastics; but, previous studies were mostly limited to a single environmental medium and did not consider the environmental conditions of the surrounding environment, so the assessment results could not truly reflect the existence of pollution in the region [18,19,20]. Therefore, in view of the above limitations, this study utilized a combination of different ecological assessment methods to evaluate the ecological risks of microplastics.

To address the gaps in the current research, this study aims to answer the following questions: What are the abundance and distribution characteristics of microplastics in different environmental media (sediment, soil, and water) and biological samples (fish and birds) in the Xisha Islands of the South China Sea? How do microplastics in environmental media and biological samples correlate, and what are the potential pathways of microplastic transfer between these compartments? What is the ecological risk posed by microplastics to the environmental media and biological populations in the Xisha Islands, and how do these risks vary across different sampling locations?

In summary, this study investigated the abundance and distribution of microplastics in different environmental media (sediment, soil, and water) and biological samples (fish and birds), analyzed the similarity of microplastics in environmental media and biological samples, and evaluated the ecological risk of microplastics on environmental media and biological samples in the South China Sea islands in view of their special geographic environments and characteristics of biological populations. The results obtained from this study will improve our understanding of microplastic contamination in the South China Sea islands and provide theoretical support for biodiversity conservation in the South China Sea islands.

## 2. Materials and Methods

### 2.1. Sample Collection

**Figure 1:**
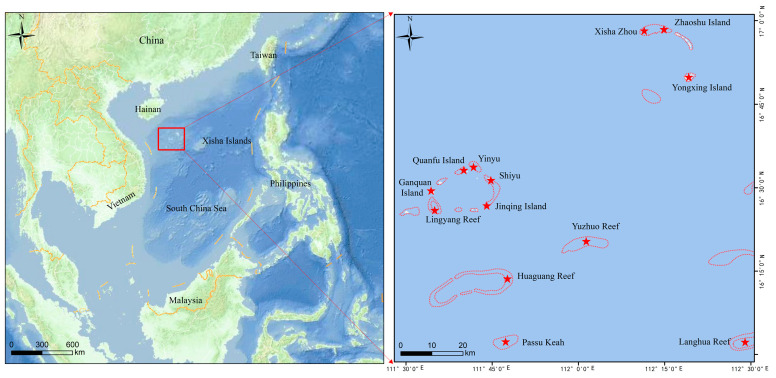
Geographic location of the sampling area of the Xisha Islands sample. The red star indicates the presence of a sampling point on that island, and the red dot line shows the approximate outline of each island.

All samples were collected from the Xisha Islands in the South China Sea (Figure 1). The sediment samples were collected on 8 April 2023. Considering the accessibility of sampling, a portion of the shallow coral reef platforms was selected for sediment collection. A total of seven sediment samples in the vicinity of the sea area of Zhaoshu, Xishazhou, and Yongxing Island were collected from 0–20 cm of the seafloor using a Peterson grab sampler (Figure S1, Table S1). Soil samples were collected on 27 September 2023. To understand the pollution status of microplastics in the Xisha Islands and to investigate whether the source of microplastics in birds is related to soil, soil samples were collected from Yongxing Island and Zhaoshu Island, which are inhabited and have relatively high levels of human activity. Five soil samples were collected randomly from 0–20 cm of the surface layer with a clean stainless steel shovel using the five-point sampling method (Figure S2, Table S2). Sediment samples were stored in brown glass bottles and soil samples were stored in aluminum foil bags. Seawater samples were collected from 29 August 2023 to 27 September 2023. Considering the impact of human activities, seawater samples were collected from the sea areas and ports where human activities are more frequent. Fourteen seawater samples around Langhua, Linyang, Huaguang, Passu Keah, and Yuzhuo Reef and Jinqing, Yinyu, Quanfu, Shiyu, Yongxing, and Zhaoshu Island were collected (Figure S3, Table S3). Approximately 1–5 L of surface seawater (50 cm below the surface) was collected using a plexiglass water collector and stored frozen in glass bottles.

Bird samples were collected in the Xisha Islands from September 2020 to June 2022; fish samples were collected in June 2022 in the areas where the bird samples were active, Ganquan Island and Zhaoshu Island, using trawl nets to catch marine fish that might be the food source of the birds. Forty-seven bird samples from four species found dead in Yongxing Island were collected. Sixty-eight fish from twenty-three species around Yongxing, Zhaoshu, and Ganquan Island were caught with the help of fishermen (Figure S4, Table S4). Upon return to the laboratory, all samples were preserved in a −20 °C refrigerator until analysis was conducted.

### 2.2. Isolation of Microplastics

#### 2.2.1. Sediment, Soil, and Water Samples

We employed the microplastic separation approach that has been extensively utilized in previous studies to separate microplastics in environmental media [21].

The frozen sediment and soil samples were thawed at room temperature and subsequently dried in an oven at 60 °C until fully dehydrated. Approximately 50–100 g of sediment sample was weighed in a long glass bottle (three parallel samples for each site), and 500 mL of saturated NaCl solution (AR, Titan, China, which had been passed through a 0.45 μm glass fiber membrane) was added. The mouth of the bottle was covered by aluminum foil, and the bottle was thoroughly stirred with a glass rod to mix the solid sediment and saturated NaCl solution into fine particles, and was left to stand for 6–12 h for flotation. The supernatant was filtered through a 0.45 μm glass fiber filter membrane, which was subsequently removed and placed flat in a glass petri dish, secured with stainless steel tweezers. The above steps were repeated two times; 50–100 mL 30% H_2_O_2_ solution (which had been passed through the 0.45 μm glass fiber filter membrane) was quickly rinsed into a conical flask. The membrane was placed in the conical flask and the conical flask was shaken every hour. The substances on the membrane were dissolved overnight (we observed whether the dissolution solution was clarified and whether there were algae and other bio-organic substances). The solution was filtered using a 0.45 μm glass fiber membrane, which was subsequently placed in an oven at 60 °C until fully dried. The cryopreserved water samples were thawed at room temperature and poured into a measuring cylinder. Their volume was recorded, and it was observed that the water samples were clarified and almost free of impurities or with very few impurities, and the water samples were filtered directly with 0.45 μm glass fiber filter membranes, which were clamped with stainless steel tweezers into petri dishes, and then placed in the oven at 60 °C until they were completely dry.

#### 2.2.2. Fish and Bird Samples

After thawing the frozen preserved fish and bird samples in a fume hood at room temperature, each fish and bird sample was weighed and recorded. The fish gills and intestines, and the stomachs of the birds, were taken out from the fish and the birds, respectively, using dissecting equipment, which was cleaned with 75% ethanol and ultrapure water. Then, the intestinal contents of fish were separated from the intestines. The mass of the fish gills, intestines, and intestinal contents and the mass of the bird stomachs were recorded.

The tissue samples after dissection were placed in a Teflon centrifuge tube. Then, 10–20 mL of mixed digestion solution (subject to complete submergence of the tissues) and proteinase K were added (the mass ratio of tissues to proteinase K was 1:1000). The compositions of the mixed digestion solution are shown in Table 1 and were prepared according to the previous study [22]. The centrifuge tubes were placed in a water bath at 60 °C for ablation for 36–48 h until complete ablation.

After complete digestion, the solution was centrifuged at 4000 rpm for 5 min, the solution was filtered through a 0.45 μm glass fiber filter membrane, and the filtered membrane was picked up with stainless steel tweezers and put into a petri dish, which was placed in an oven at 60 °C until it was completely dry.

### 2.3. Observation and Identification of Microplastics

The filter membrane obtained from the above steps was placed under a stereomicroscope (Axiocam 208 color, ZEISS, BW, Oberkochen, Germany); based on the physical properties described in previous studies for visual identification, microplastics were quantified and classified. The suspected microplastics were picked out using pointed tweezers. The imaging system with the microscope was used to take pictures and measure the particle size of the suspected microplastics, as well as record the color, shape, and other physical characteristics.

The suspected microplastics that were picked were placed on the carrier stage of the Fourier Transform microinfrared spectrometer (FTIR; LUMOS II, BRUKER, Billerica, MA, USA) for detection. Depending on the size and thickness of the suspected microplastics, transmission, reflectance, or ATR modes were selected, and the spectra were scanned 64 times. The spectra were analyzed at a resolution of 4 cm^−1^ and in the spectral range of 600–4000 cm^−1^, and the resulting spectra were matched with a library of polymer spectra. The identification results were recorded as the material of the microplastics, and their abundance was finally calculated.

### 2.4. Quality Control of Experiments

Strict controls are required to prevent exogenous contamination during sample collection, pre-treatment, and identification. The glassware used in the analysis was rinsed three times with ultrapure water. All solutions used in the analysis were filtered through a 0.45 μm glass fiber membrane. The samples and glassware were covered with aluminum foil to avoid contact with the air during placement. A blank control group was included in each batch of samples, and the abundance of microplastics detected in the blank control group was 0, indicating that the exogenous contamination of the samples during the pre-treatment process was negligible.

### 2.5. Ecological Risk Assessment

At present, a standardized system for assessing the ecological risk of microplastics is lacking. In this study, we summarized three commonly used indicators for evaluating the ecological risk of microplastics concerning the microplastic ecological risk assessment methods used by the previous researchers, which mainly include pollution load index (PLI), polymer hazard index (PHI), and potential ecological risk index (PERI). These three indices are mainly based on microplastic abundance, polymer toxicity, and the percentage of each component [23].

The pollution load index, which mostly depends on pollutant concentrations, was initially proposed by Tomlinson to assess the pollution of heavy metals in the environment [24]. Among them, the selection of background values is more important:

(1) $$C_{F}=C/C_{0}$$

(2) $$PLI=\sqrt{C_{F}}$$

(3) $${PLI}_{zone}=\sqrt[{n}]{{PLI}_{1}\times {PLI}_{2}\times {PLI}_{3}\times \ldots \times {PLI}_{n}}$$

where *C* is the microplastic abundance in the sample (items/kg, items/L or items/ind), *C*_0_ is the background value (items/kg, items/L or items/ind), and *PLI* is the microplastic pollution load index of the sample. The parameter *C*_0_ is usually chosen as the background value for the smallest value of microplastic abundance in similar samples from previous or current studies. This study chose a background value of 10 items/kg for seawater sediment, 2.2 items/kg for soil, 0.08 items/L for seawater, 0.03 items/ind for fish samples, and 1.31 items/ind for bird samples [25].

In addition to the abundance of microplastics, polymer type is a critical characteristic that affects their toxicity. Lithner et al. modelled risk levels based on risk class and category and calculated polymer risk scores based on monomer hazards [26]:

(4) $$PHI=\sum_{n=1}^{n}P_{n}S_{n}$$

where *Pn* is the proportion of polymers in each sample (%) and *Sn* is the risk score corresponding to the type of micro-plasticized polymer. This study set the *Sn* value to 1 for rayon and other unclassified polymers.

The potential ecological risk index (PERI) is based on the measured abundance of microplastics in the study area, combined with the hazard coefficients of the various microplastics, to derive the potential risk level of microplastics for site and regional contamination:

(5) $$T_{i=}\sum_{n=1}^{n}{(P}_{n}\times S_{n})$$

(6) $${PERI}_{i}=T_{i}\times C_{i}/C_{0}$$

where *Pn* represents the percentage of microplastic species n in sample *i* (%), *Sn* represents the toxicity risk index of microplastic species *n*, *n* represents the number of microplastic polymer species in sample *i*, *T_i_* represents the integrated toxicity response factor of microplastics in a sample, *C_i_* is the abundance of microplastics in sample *i* (items/kg, items/L or items/ind), *C*_0_ is the environmental safety concentration (items/kg, items/L or items/ind), and *PERI_i_* is the integrated potential ecological risk index of sample *i*. In this study, the safe environmental concentration of microplastics in sediment was 540 items/kg, the safe concentration of microplastics in soil was 118.8 items/kg, the safe concentration of microplastics in seawater was 6.65 items/L, the safe concentration of microplastics in fish was 12.1 items/ind, and the safe concentration of microplastics in birds was 22.8 items/ind [27].

### 2.6. Data Analysis

IBM SPSS 26 was applied to analyze the data, and the Shapiro–Wilk (S-W) test was used to test for conformity to normal distribution before data analysis. Neither the raw data nor the log-transformed data conformed to normal distribution, and the differences in microplastic abundance between different sampling points of environmental samples and other species of biological samples were analyzed using the nonparametric Kruskal–Wallis test. Spearman’s correlation analysis was used to analyze the correlation between the abundance of microplastics in different samples and between the body weights of the birds and the abundance of microplastics, and the statistical significance level was set at *p* < 0.05. The graphs were plotted using Origin 2022 and ArcGIS 10.2.

In this study, the abundance of microplastics in sediment and soil was calculated as the number of microplastics detected per kg of dry weight sample (items/kg); the abundance of microplastics in seawater was calculated as the number of microplastics detected per L of sample (items/L); and the abundance of microplastics in fish and birds was calculated as the number of microplastics detected in each sample (items/ind).

## 3. Results and Discussion

### 3.1. Abundance of Microplastic Pollution

#### 3.1.1. Abundance of Microplastic in Environmental Media

Microplastics were detected in all environmental media samples (Table 2), for which there were average abundances of 41.56 ± 19.12 items/kg, 92.94 ± 111.05 items/kg, and 2.89 ± 1.92 items/L for sediments, soils, and water, respectively. This result indicated that microplastics are prevalent in the environmental media of the Xisha Islands.

The abundance of microplastics in sediment and seawater from the Xisha Islands were 52.61–190.55 items/kg and 0.2–45.2 items/L, respectively [2]. The average abundance of microplastics in the soils of Yongxing and Dongdao Islands were 512 ± 110 items/kg and 219 ± 45 items/kg in a previous study [28]. The microplastic abundance in sediments, soil, and water samples in the same area reported in previous studies was higher than those in the present study.

The concentrations of microplastics detected in the sediments of the Bohai Sea, the Yellow Sea, and the Black Sea were 41.10–67.63 items/kg, 560–4205 items/kg, and 106.7 items/kg, respectively [13,14,29]. These abundances were also higher than those in the South China Sea in the present study. However, compared with the seawater of the Western Pacific Ocean (240 items/m^3^) and the Jiaozhou Bay (20–120 items/m^3^), the abundances of microplastics in the South China Sea were higher [16,30].

One of the dominant factors causing this difference is the various sampling and detection methods used in different studies, which will have a significant impact on the detection of microplastics and the recording of microplastic types, sizes, and abundances [31], resulting in potentially large variations in the abundance of microplastics reported in different studies. The minimum particle size of microplastics detected in the Bohai Sea, Yellow Sea, and Black Sea was 0.03 mm, 0.05 mm, and 0.02–0.03 mm, respectively, and this is 0.1 mm for the West Pacific Ocean and Jiaozhou Bay, while the minimum particle size in this study was 0.09 mm. Previous studies identified small-sized microplastics as predominant, which explains why the abundance of microplastics detected in this study was lower than that in the Bohai Sea, the Yellow Sea, the Black Sea, and other seas, and higher than that in the West Pacific Ocean and Jiaozhou Bay.

#### 3.1.2. Abundance of Microplastic in Biological Samples

Microplastics were detected in the bodies of 56 out of 68 fish samples, with a total detection rate of 82.4% and an average abundance of 2.57 ± 2.12 items/ind (Table 3), indicating that microplastic contamination is prevalent in fish in the South China Sea. Among them, the highest abundance of microplastics was detected in the *Upeneus sulphureus* (5 ± 2.83 items/ind), while no microplastics were detected in fish such as *Coris gaimard*, *Branchiostegus japonicus*, and *Neoniphon opercularis*. The abundance of microplastics detected in fish samples in the present study was at the same range of those reported in fish from the Xisha Islands (0–12 items/ind) by Ding et al. [2]. Current studies on microplastics in fish indicate that microplastic ingestion by fish is widespread in natural marine areas and tends to vary among different marine areas and populations [32].

Microplastics were detected in 32 out of 47 bird samples, with a detection rate of 68.1%. The abundance of microplastic ranged from 0 items/ind (*Ardea purpurea*) to 3.5 items/ind (*Egretta garzetta)* (Table 3), with an average abundance per individual of 1.702 ± 1.50 items/ind. This abundance was at the same level as that detected in the feces of birds from Yancheng, Jiangsu Province (1.5 ± 0.87~3.4 ± 1.50 items/ind) [33], but was lower than that in birds from the Yellow River Delta (4.45 ± 0.11 items/ind) [34] and birds from the state of Florida, USA (11.9 ± 2.8 items/ind) [35].

Some studies have found that waterbirds are at a high risk of exposure to and ingestion of microplastics because of their prolonged migratory patterns and frequent congregation along shorelines, coastal regions, and estuaries, which are characterized by elevated plastic pollution levels. The birds’ uptake of microplastics may be dependent on vision (color or shape) or touch, so the size and shape of microplastics, as well as their texture, are key determinants of their uptake [36].

In addition, we evaluated the correlation between the abundance of microplastics on different islands and their distance from the mainland. The research results indicated that there was no significant correlation between the abundance of microplastics on different islands and their distance from the mainland. The possible reason is that the microplastic pollution of each island is mainly related to human activities on the island, and is less affected by the ocean currents from the mainland.

### 3.2. Characteristics of Microplastics in Environmental Media and Biological Samples

**Figure 2:**
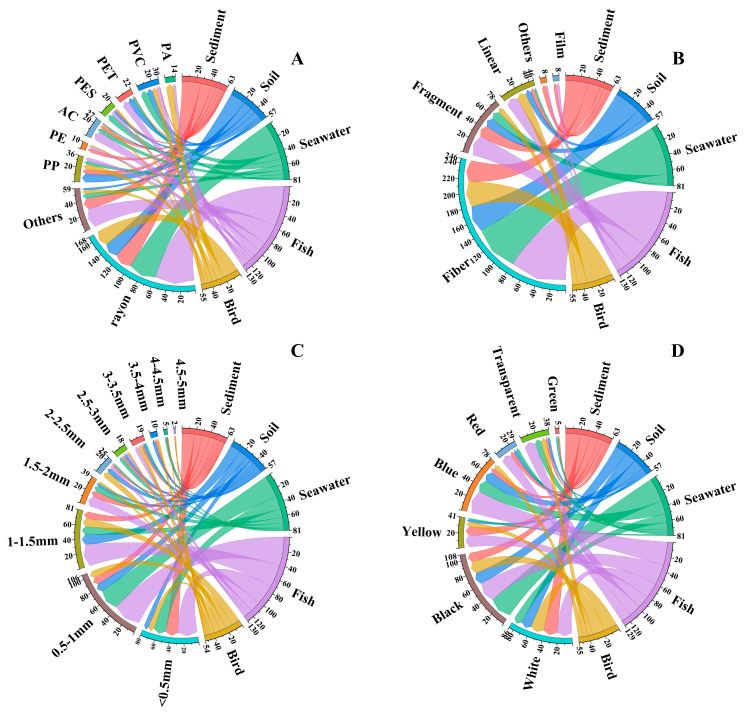
Microplastic characterization of environmental media (sediment, soil, and water) and biological samples (fish and bird). Microplastic material composition (**A**), microplastic shape composition (**B**), microplastic particle size composition (**C**), microplastic color composition (**D**).

This study identified microplastic components in environmental and biological samples, primarily consisting of rayon, PP, PET, AC, PVC, PA, PES, and PE (as shown in Figure 2A; the FTIR spectra are shown in Figure S5). Rayon was the dominant microplastic material detected in both environmental samples (sediment, soil, and seawater) and biological samples (fish and birds), with the percentages of 41.3%, 38.6%, 50.6%, 46.3%, and 40.6%. Rayon, as one of the oldest commercially available man-made fibers, is made from natural cellulose (usually from wood pulp) through chemical processing, and combines the comfort of natural fibers with the durability and malleability of synthetic fibers. It is mainly used in personal hygiene products and textiles [37,38]. Due to its low crystallinity and orientation, rayon products can easily and rapidly crack [39], which explains the difference between environmental samples and biological samples. In addition, most of the rayon exists in the form of fibers, and since the Xisha Islands are located in the tropics, high temperatures will exacerbate the aging rate of the fibers, leading to an increase in the amount of rayon detected [40]. This also explains the high detection rate of rayon in environmental and biological samples.

Fibers were the dominant microplastic shape in both environmental (sediment, soil, and seawater) and biological samples (fish and birds) (as shown in Figure 2B; the main shapes of the detected microplastics are shown in Figure S6), accounting for 47.6%, 63.2%, 81.5%, 60.6%, and 68.8%, respectively. Similar to our findings, fibers were also the most common shape detected in most of the previous studies, mainly from textile production, domestic wastewater discharges, and the airborne deposition of microplastics [41]. The high detection rate of rayon materials and fiber shapes in environmental samples may be related to the high fish farming activities in the coastal areas of the South China Sea, where fibers are easily released when textile materials such as fishing nets are shed, as well as domestic sewage discharges, which also produce large quantities of fibers [42,43]. In addition to the high generation rate of fibers in coastal fishery areas, the high detection rate of rayon materials and fiber shape in biota may also be due to their high bioaccumulate rate in organisms. Fibers may become entangled with each other to form clumps and, thus, affect the physiology of organisms by blocking the digestive tract rather than with particles and fragments of microplastics [44]. The impacts of fibrous microplastics on the marine environment cannot be ignored.

The particle size of microplastics detected in sediments was mainly < 0.5 mm, accounting for 28.6%; in seawater and fish, this was mainly 0.5–1 mm, accounting for 39.5% and 33.7%, respectively; and in soil and birds, this was mainly 1–1.5 mm, accounting for 26.3% and 24.6%, respectively (as shown in Figure 2C). There are two main ways for microplastics to be ingested in biological samples: one is directly from the external environment, and the other is indirectly from other organisms through predation, and the direct ingestion of microplastics from the external environment is the most important source of microplastics in organisms [45]. Birds inadvertently consume microplastics from terrestrial ecosystems during predation, whereas the primary source of microplastics in fish is from seawater [2]. Consequently, the particle size distribution of microplastics detected in soil and birds were similar, and the particle size distribution of microplastics detected in seawater and fish were similar.

The color of microplastics detected in environmental and biological samples were slightly different: those in sediment were predominantly white, with a percentage of 31.7%; those in soil and seawater were predominantly black, with a percentage of 29.8% and 40.7%, respectively; those in fish were predominantly blue, with a percentage of 21.7%; and those in birds were predominantly black and white, with a percentage of 32.8% (as shown in Figure 2D). It is noteworthy that only green microplastics were detected in sediment and seawater samples, and it was found that most microplastics in sediment entered from seawater, and smaller microplastics may sink to the seafloor by attaching to feces or marine snow [16,46]. Therefore, there is some similarity between microplastics in sediment and seawater. Red microplastics were detected in all five samples except the bird sample, and this study speculated that birds may have some selectivity for the color of microplastics as they avoid red particles (the contamination characteristics of microplastics detected in various environmental media and biological samples are shown in Figures S7–S11).

**Figure 3:**
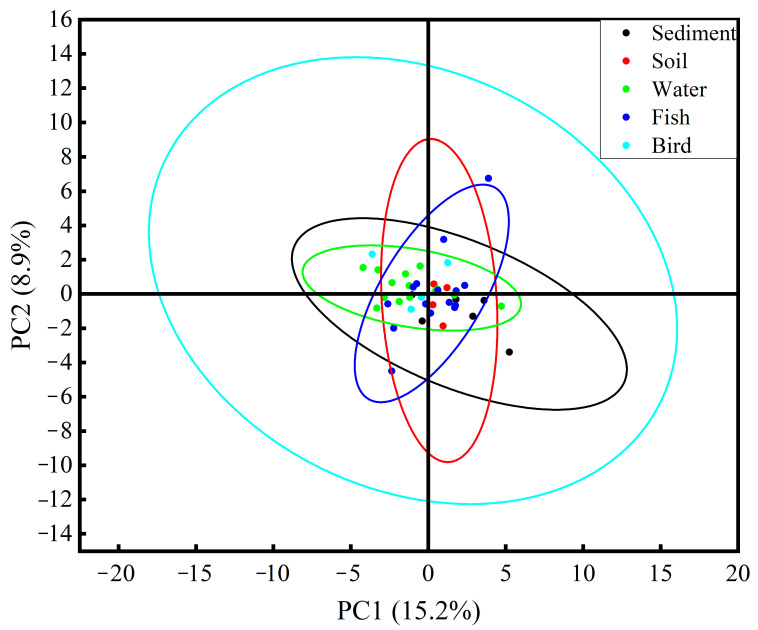
PCA analysis of microplastic contamination characteristics detected in environmental media (sediment, soil, and water) and biological samples (fish and bird).

Principal component analysis (PCA) was performed to investigate the differences in microplastic characteristics detected in environmental media and biological samples (Figure 3). The results of the study showed that there was no significant difference in microplastic characteristics between environmental media and biological samples, and that environmental media and biological samples possessed similar sources of microplastics. This indicated that wild organisms in the Xisha Island area feed over a wide area and, therefore, microplastics detected in the body are very close to those detected in environmental media, with fish samples being closer to the environmental media (the PCA analysis of microplastic contamination characteristics detected in different environmental media and in different islands is shown in Figures S12 and S13). In addition, PCA can also be combined with neural networks to analyze the sources and correlations of pollutants [47].

### 3.3. Ecological Risk Assessment

The PLI values of the sediment, soil, seawater, fish, and bird were 1.94, 5.20, 5.21, 9.37, and 1.26, respectively. Based on the PLI, the microplastic pollution in the Xisha Islands was in risk class I (Table S5). It is worth noting that the PLI model assessed the ecological risk based on the abundance of microplastics without considering the potential toxicity of different polymer types. Therefore, despite the low level of microplastic contamination estimated by the PLI model, it did not mean actual low risks [48].

If we consider the polymer types, the microplastic contamination in the Xisha Islands was in risk class III (Table S6), with PHIs of 162.51, 719.95, 691.92, 331.85, and 745.05 for five samples: sediment, soil, seawater, fish, and bird, respectively. The reason for the high-risk class of microplastic contamination in the Xisha Islands may be the presence of high-toxicity-coefficient polymers, such as PVC (5001) and Arcylic (1021) (Table S7). PVC is widely used among common items such as building materials, artificial leather, pipes, wires and cables, packaging films, etc. [23], and Arcylic is an important malleable polymer with a wide range of applications in the construction industry. These polymers usually have a high density, and may, therefore, penetrate deep into various water layers of the ocean and cause unknown impact in the deep sea. Therefore, regulating PVC and Arcylic inputs will be an effective way to reduce the overall risk of microplastics in the Xisha Islands in the future.

**Figure 4:**
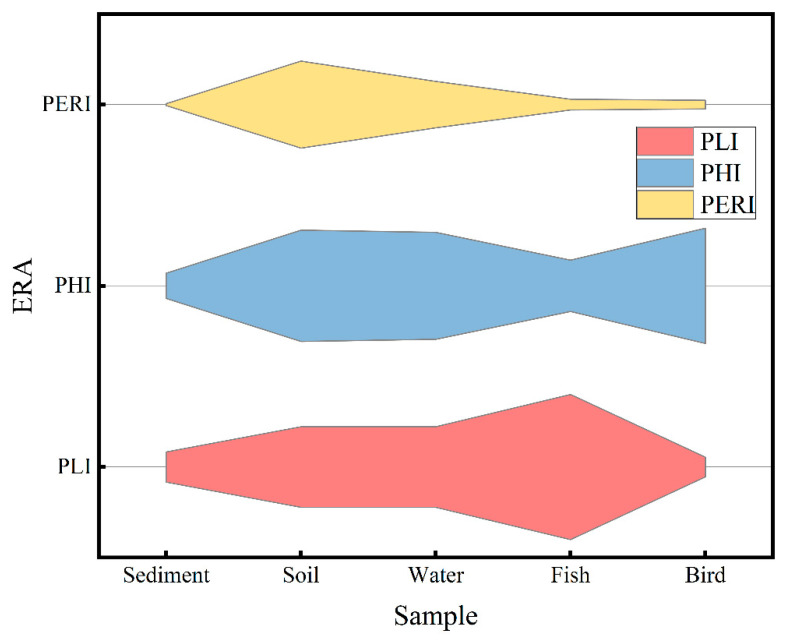
Ecological risk assessment (pollution load index (PLI), polymer hazard index (PHI), and potential ecological risk index (PERI)) of microplastics in five types of samples (sediment, soil, water, fish, bird).

If considering both abundance and types, the PERI values for the five samples of sediment, soil, seawater, fish, and bird were 12.51, 563.22, 300.51, 79.75, and 71.17, respectively. Except for the microplastic pollution detected in soil and seawater, which was at medium risk, the microplastic pollution in the other samples was at no risk (Table S8). Some studies have derived an environmentally safe abundance of 6650/m^3^ [27] and a biological no-effect abundance of 12,000/m^3^ [49], below which adverse effects are unlikely to occur, and no direct impacts are expected from free-floating microplastics in the marine environment until the year 2100. However, even today, it is possible to exceed safe concentrations where microplastic contamination is high. The three different ecological risk assessment methods yielded completely different conclusions due to the other factors they considered (PHI is influenced by microplastic abundance, PLI is influenced by microplastic type, and PERI considers both abundance and type of microplastics, Figure 4), which will directly affect the formulation of related policies. Therefore, there is an urgent need to establish standard ecological risk assessment methods to increase our understanding of the status of microplastic pollution.

## 4. Conclusions

In this study, the abundance, distribution, and pollution characteristics of microplastics in the Xisha Islands, South China Sea, were analyzed, and an ecological risk assessment was conducted. The results showed that the abundance of sediment, soil, seawater, fish, and birds was 41.56 ± 19.12 items/kg, 92.94 ± 111.05 items/kg, 2.89 ± 1.92 items/L, 2.57 ± 2.12 items/ind, and 1.702 ± 1.50 items/ind, respectively. There was a large spatial heterogeneity in the abundance of microplastics, which ranged from 0.97 to 7960 items/kg in sediments and between 0.2 × 10^−5^ and 5.9 items/L in surface seawater, and the detection rate of microplastics in fish in other marine areas ranged from 0.25% to 100%. Compared with other studies, microplastic contamination in the sediments of the Xisha Islands, South China Sea, detected by this study, was at a low level, whereas microplastic contamination in other environmental media and biological samples were in the middle-to-high level. The microplastics detected in the five types of samples were all rayon-dominated and fiber-dominated in shape. The color of microplastics detected in sediments was mainly white, in soil and seawater it was mainly black, in fish it was mainly blue, and in birds it was mainly black and white. The particle size of microplastics detected in sediments was mainly <0.5 mm, while that in seawater and fish was mainly 0.5–1 mm, and in soil and birds it was mainly 1–1.5 mm. The widespread presence of microplastics in the Xisha Islands may simply stem from the discharge of personal hygiene products, textiles, washing wastewater, and well-developed fish farming. By evaluating the PLI, PHI, and PERI, the PLI of the Xisha Islands indicated that the hazard level was slightly polluted, the PHI was high-risk, and the PERI of microplastics in the remaining samples was in the no-risk level, except for the PERI of the soil and seawater, which was in the medium-risk level. Compared with PLI and PHI, which determine ecological risk based on only one influencing factor, PERI may be more accurate as it considers the abundance of microplastics and the type of microplastics.

Overall, based on the microplastic characteristics and risk assessment, we suggest that the monitoring of rayon, PVC, and Arcylic should be emphasized in the subsequent research. This study provided a solid theoretical basis for the risk assessment of microplastics in the Xisha Islands, where the potential risk of microplastic pollution in the ecosystem is not only related to the content and composition of microplastics, but also to their physicochemical properties. Future studies should pay more attention to these aspects of microplastics in order to comprehensively assess the overall risk of microplastic pollution to marine ecosystems and human health.

## Figures and Tables

**Table 1:** Composition table of mixed digestion solution.

Ingredient	Quantity Contained
Tris-HCl	400 mmol/L
EDTA-2Na	60 mmol/L
NaCl	105 mmol/L
SDS	10 g
H_2_O	1 L

**Table 2:** Microplastic abundance in environmental media.

Sediment		Soil		Seawater	
Sampling Point *	Abundance (items/kg)	Sampling Point *	Abundance (items/kg)	Sampling Point *	Abundance (items/L)
Se1	23.68 ± 8.19	S1	288.21 ± 170.65	W1	7
Se2	53.06 ± 41.39	S2	23.79 ± 29.71	W2	0.99
Se3	49.71 ± 0.26	S3	41.64 ± 14.41	W3	1.11
Se4	23.60 ± 8.19	S4	33.31 ± 21.82	W4	2.94
Se5	20.47 ± 10.15	S5	77.74 ± 19.19	W5	2.97
Se6	70.55 ± 29.16			W6	4.81
Se7	49.86 ± 33.01			W7	3.77
				W8	4.69
				W9	4.32
				W10	3.21
				W11	0.46
				W12	0.41
				W13	2.11
				W14	1.62

* The specific information of the sampling point can be found in the Supplementary Materials (Figures S1–S3, Tables S1–S3).

**Table 3:** Microplastic abundance in biological samples.

Fish		Birds	
Fish Species	Abundance (items/ind)	Bird Species	Abundance (items/ind)
*Epinephelus chlorostigma*	2.44 ± 2.60	*Ardea intermedia*	1.31 ± 1.47
*Amphilophus*	4.25 ± 0.96	*Egretta garzetta*	3.5
*Lethrinus ornatus*	2.80 ± 1.92	*Ardea cinerea*	2
*Datnioides microlepis*	7	*Ardea purpurea*	0
*Acanthurus triostegus*	2 ± 2.65		
*Acanthurus nigrofuscus*	1.6 ± 1.52		
*Scarus psittacus*	2 ± 1.41		
*Scarus dimidiatus*	2 ± 1.41		
*Coris gaimard*	0		
*Upeneus sulphureus*	5 ± 2.83		
*Pterocaesio tile*	5 ± 1.41		
*Parupeneustrifasciatus*	3 ± 2.55		
*Cephalopholis urodeta*	2 ± 2.35		
*Sufflamen fraenatus*	1		
*Cheilinus rhodochrous Gunther*	2 ± 1.41		
*Malacanthus brevirostris*	2.4 ± 1.67		
*Abalistes stellatus*	3.67 ± 3.06		
*Branchiostegus japonicus*	0		
*Sparus fasciatus*	2		
*Sufflamen chrysopterus*	2.33 ± 0.58		
*Neoniphon opercularis*	0		
*Hologymnosus doliatus*	3		
*Hapalogenys mucronatus*	2.67 ± 2.52		

## Data Availability

The data are contained within the article.

## Supplementary Materials

The following supporting information can be downloaded at: https://www.mdpi.com/article/10.3390/toxics13030205/s1. Figure S1. Map of sediment sampling locations; Table S1. Information on sediment sampling locations; Figure S2. Map of soil sampling locations; Table S2. Information on soil sampling locations; Figure S3. Map of seawater sampling locations; Table S3. Information on seawater sampling locations; Figure S4. Map of biological sampling locations; Table S4. Information on the quantity of biological samples; Figure S5. FTIR profile of major polymers in microplastics in the Paracel Islands in the South China Sea; Figure S6. Shape of microplastics in the Paracel Islands in the South China Sea; Figure S7. Characterization of microplastic contamination in sediments: microplastic material composition and number of microplastics at each sampling site (A), microplastic shape composition and number of microplastics at each sampling site (B), microplastic color composition and number of microplastics at each sampling site (C), microplastic particle size composition and number of microplastics at each sampling site (D); Figure S8. Characterization of microplastic contamination in soil: composition and number of microplastic materials at each sampling point (A), composition and number of microplastic shapes at each sampling point (B), composition and number of microplastic colors at each sampling point (C), composition and number of microplastic particle sizes at each sampling point (D); Figure S9. Characteristics of microplastic pollution in seawater: composition and number of microplastic materials at each sampling point (A), composition and number of microplastic shapes at each sampling point (B), composition and number of microplastic colors at each sampling point (C), and composition and number of microplastic particle sizes at each sampling point (D); Figure S10.Characterization of microplastic contamination in fish samples: microplastic material composition and number of microplastics per species (A), microplastic shape composition and number of microplastics per species (B), microplastic color composition and number of microplastics per species (C), microplastic particle size composition and number of microplastics per species (D); Figure S11. Characterization of microplastic contamination in bird samples: microplastic material composition and number of microplastics per species (A), microplastic shape composition and number of microplastics per species (B), microplastic color composition and number of microplastics per species (C), microplastic particle size composition and number of microplastics per species (D); Figure S12. PCA characterization of microplastic contamination detected in different environmental media; Figure S13. PCA analysis of microplastic contamination characteristics detected in different island environmental media; Table S5. Classification of microplastic pollution load index levels; Table S6. Microplastic risk index rating scale; Table S7. Microplastic hazard score values; Table S8. Classification of potential ecological risk indices for microplastics.
