# Supplementary material for: Microplastic Pollution and Its Ecological Risks in the Xisha Islands, South China Sea
Source: Toxics. 2025 Mar 12;13(3):205. doi: 10.3390/toxics13030205 (PMC11946236; doi:10.3390/toxics13030205)
Supplement: Supplementary file 1 [file toxics-13-00205-s001.zip › toxics-3505755-supplementary.pdf]

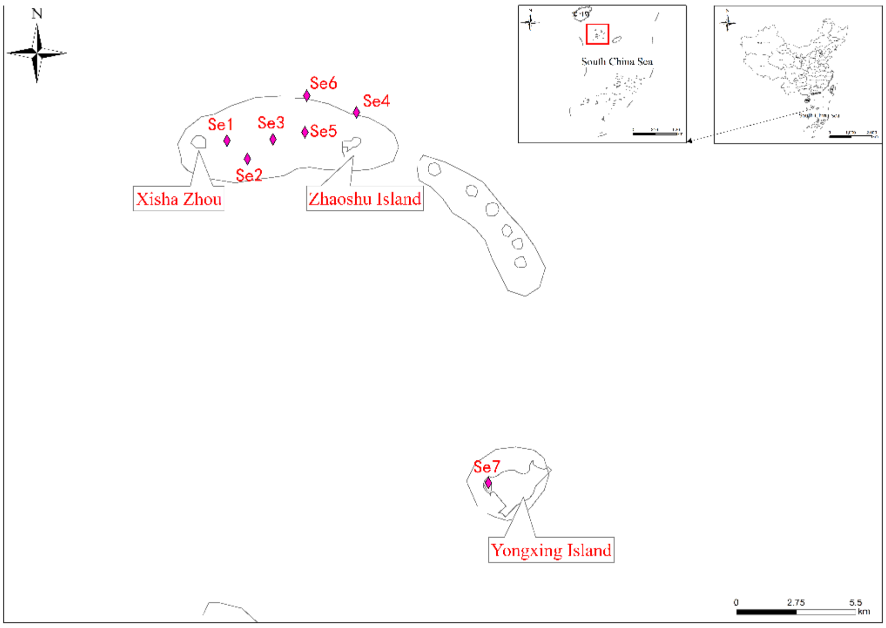

Figure S1. Map of sediment sampling locations

Table S1. Information on sediment sampling locations

Sampling point	Sampling area	Latitude and Longitude
Se1	Zhaoshu Island and Xishazhou	112.2162028E,16.9808583N
Se2		112.2250472E,16.9732222N
Se3		112.2361528E,16.9813139N
Se4		112.2723417E,16.9923111N
Se5		112.2500083E,16.9841722N
Se6		112.2508917E,16.9994000N
Se7	Yongxing Island	112.3284000E,16.8376000N

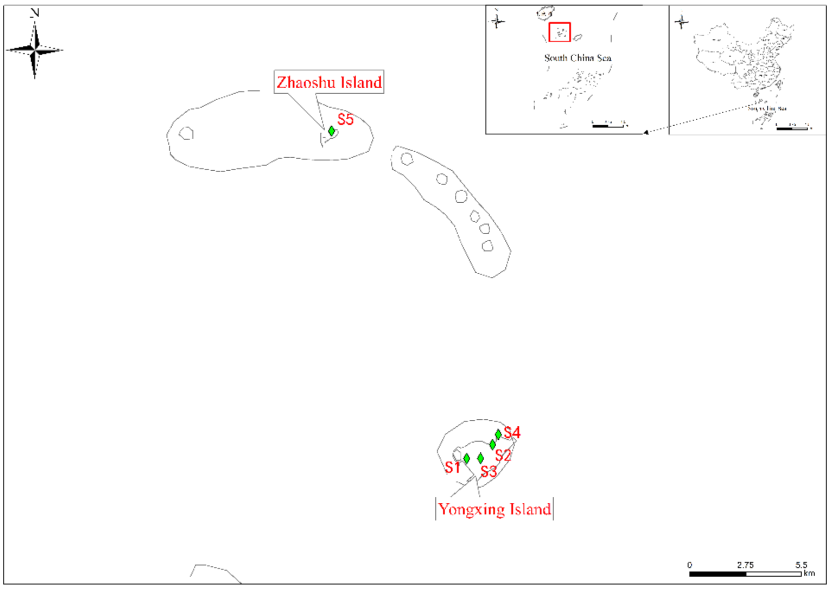

Figure S2. Map of soil sampling locations

Table S2. Information on soil sampling locations

Sampling point	Sampling area	Latitude and Longitude
S1		112.3325879E,16.8350169N
S2	Yongxing Island	112.3445795E,16.8411098N
S3		112.3390266E,16.8350742N
S4		112.3473006E,16.8456142N
S5	Zhaoshu Island	112.2711614E,16.9810315N

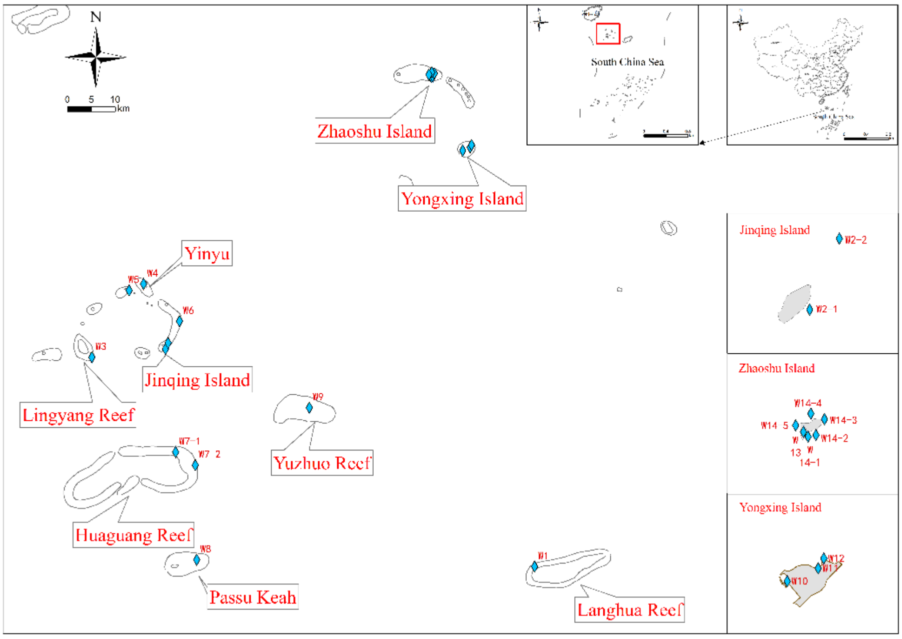

Figure S3. Map of seawater sampling locations

Table S3. Information on seawater sampling locations

Sampling point	Sampling area	Latitude and Longitude
W1	Langhua Reef	112.4651813E,16.0466414N
W2-1*	Jinqing Island	111.7440600E,16.4623853N
W2-2*		111.7491352E,16.4739920N
W3	Linyang Reef	111.5996715E,16.4471152N
W4	Yinyu	111.7015385E,16.5856678N
W5	Quanfu Island	111.6732339E,16.5735850N
W6	Shiyu	111.7717655E,16.5149007N
W7-1*	Huaguang Reef	111.7632200E,16.2672080N
W7-2*		111.8016154E,16.2423083N
W8	Passu Keah	111.8037678E,16.0630368N
W9	Yuzhuo Reef	112.0257183E,16.3500990N
W10	Yongxing Island	112.3298228E,16.8352966N
W11		112.3445795E,16.8411098N

Sampling point	Sampling area	Latitude and Longitude
W12	Zhaoshu Island	112.3473006E,16.8456142N
W13		112.2672859E,16.9770953N
W14-1*		112.2690172E,16.9753714N
W14-2*		112.2719725E,16.9759433N
W14-3*		112.2752453E,16.9816809N
W14-4*		112.2701257E,16.9837744N
W14-5*		112.2643426E,16.9794930N

*W2, W7, and W14 are samples formed by mixing two or more sampling points into one.

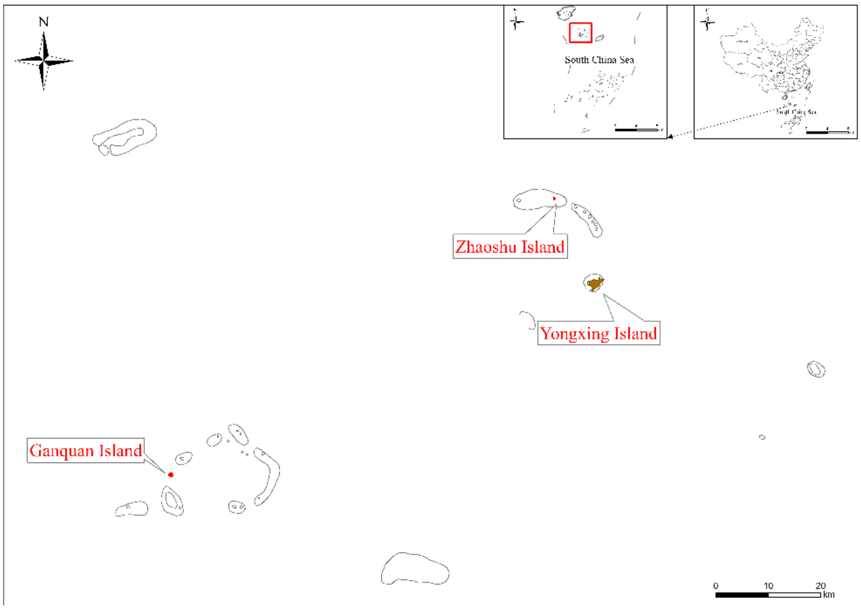

Figure S4. Map of biological sampling locations

Table S4. Information on the quantity of biological samples

Fish		Birds	
Fish species	Individual quantity	Birds species	Individual quantity
Epinephelus chlorostigma	9	*Ardea intermedia*	42
Amphilophus	4	*Egretta garzetta*	2
Lethrinus ornatus	5	*Ardea cinerea*	1
Datnioides microlepis	1	*Ardea purpurea*	2
Acanthurus triostegus	3		
Acanthurus nigrofuscus	5		

Fish		Birds	
Fish species	Individual quantity	Birds species	Individual quantity
Scarus psittacus	2		
Scarus dimidiatus	2		
Coris gaimard	1		
Upeneus sulphureus	2		
Pterocaesio tile	2		
Parupeneustrifasciatus	5		
Cephalopholis urodeta	5		
Sufflamen fraenatus	1		
Cheilinus rhodochrous Gunther	2		
Malacanthus brevirostris	5		
Abalistes stellatus	3		
Branchiostegus japonicus	2		
Sparus fasciatus	1		
Sufflamen chrysopterus	3		
Neoniphon opercularis	1		
Hologymnosus doliatus	1		
Hapalogenys mucronatus	3		
Total	68		47

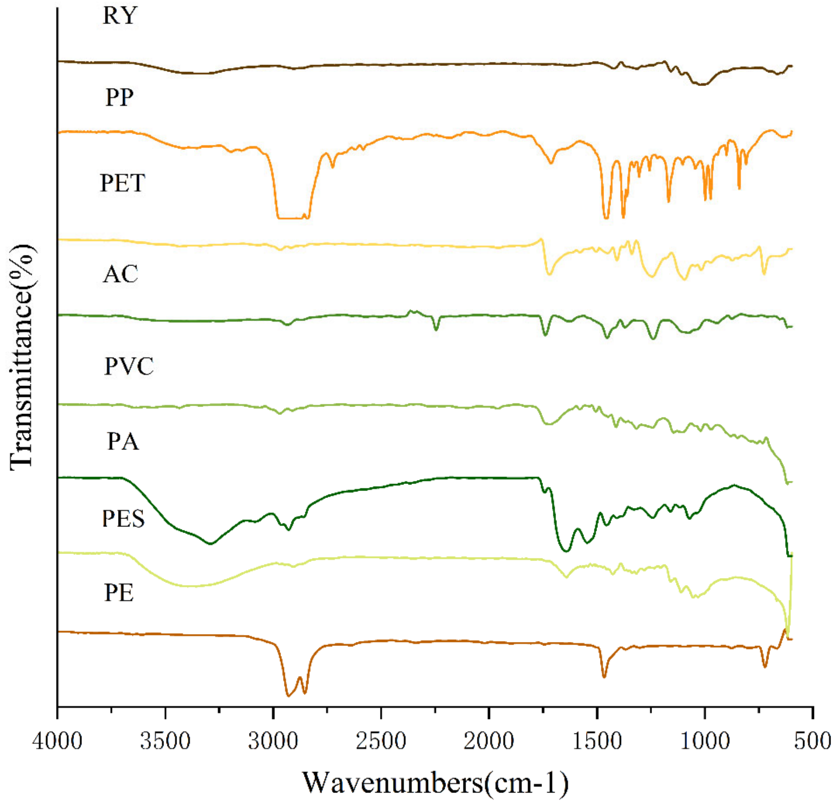

Figure S5. FTIR profile of major polymers in microplastics in the Paracel Islands in the South China Sea

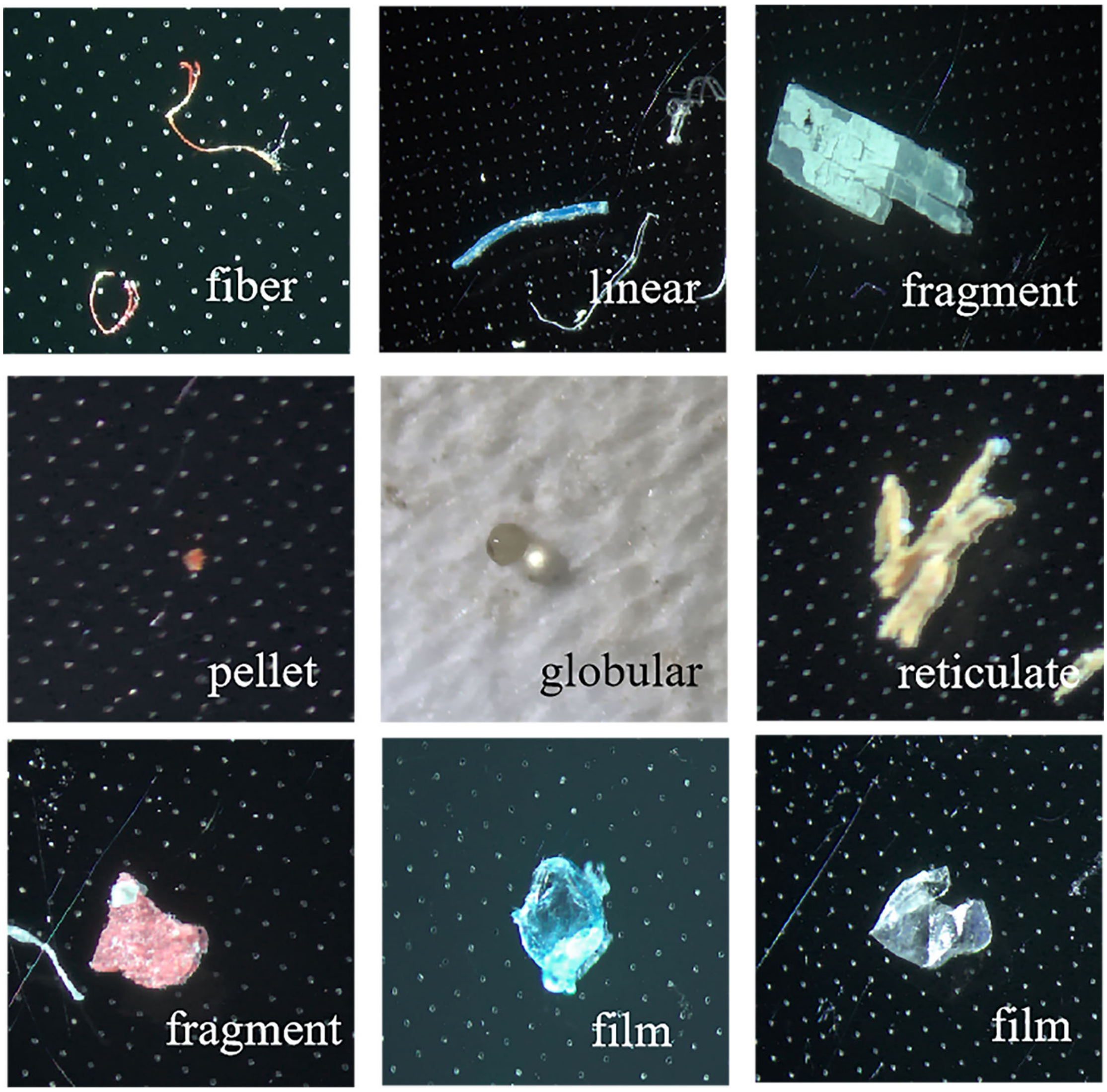

Figure S6. Shape of microplastics in the Paracel Islands in the South China Sea

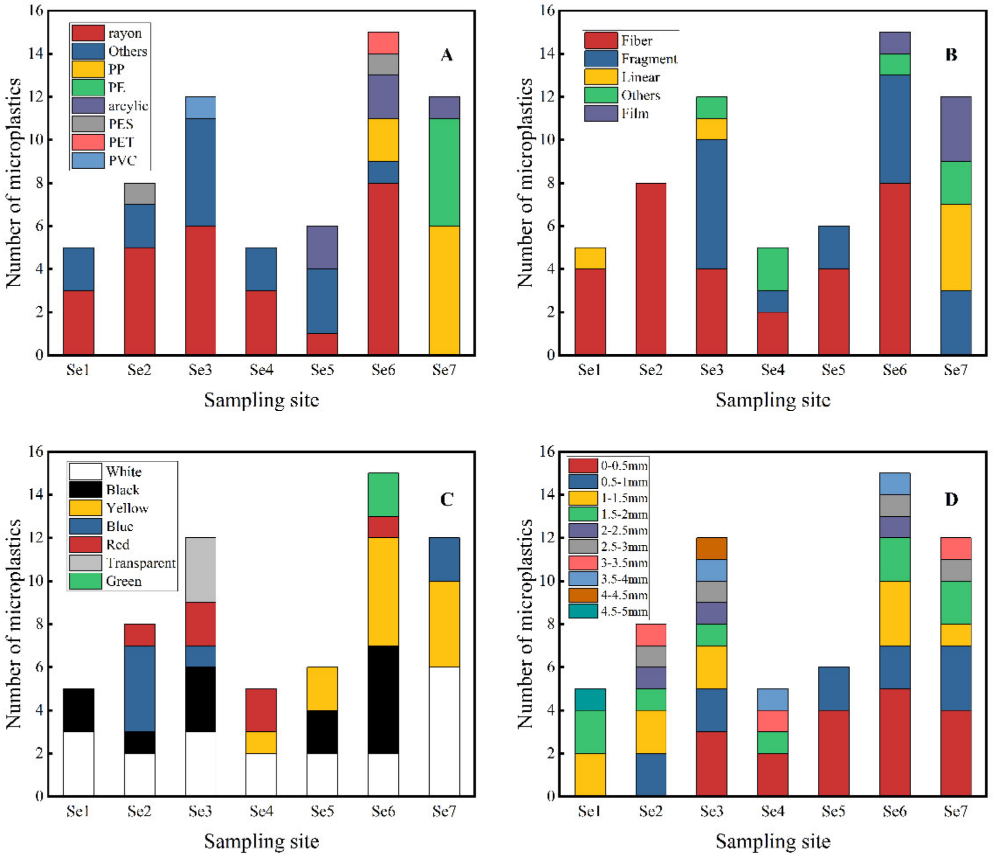

Figure S7. Characterization of microplastic contamination in sediments, microplastic material composition and number of microplastics at each sampling site (A), microplastic shape composition and number of microplastics at each sampling site (B), microplastic color composition and number of microplastics at each sampling site (C), microplastic particle size composition and number of microplastics at each sampling site (D)

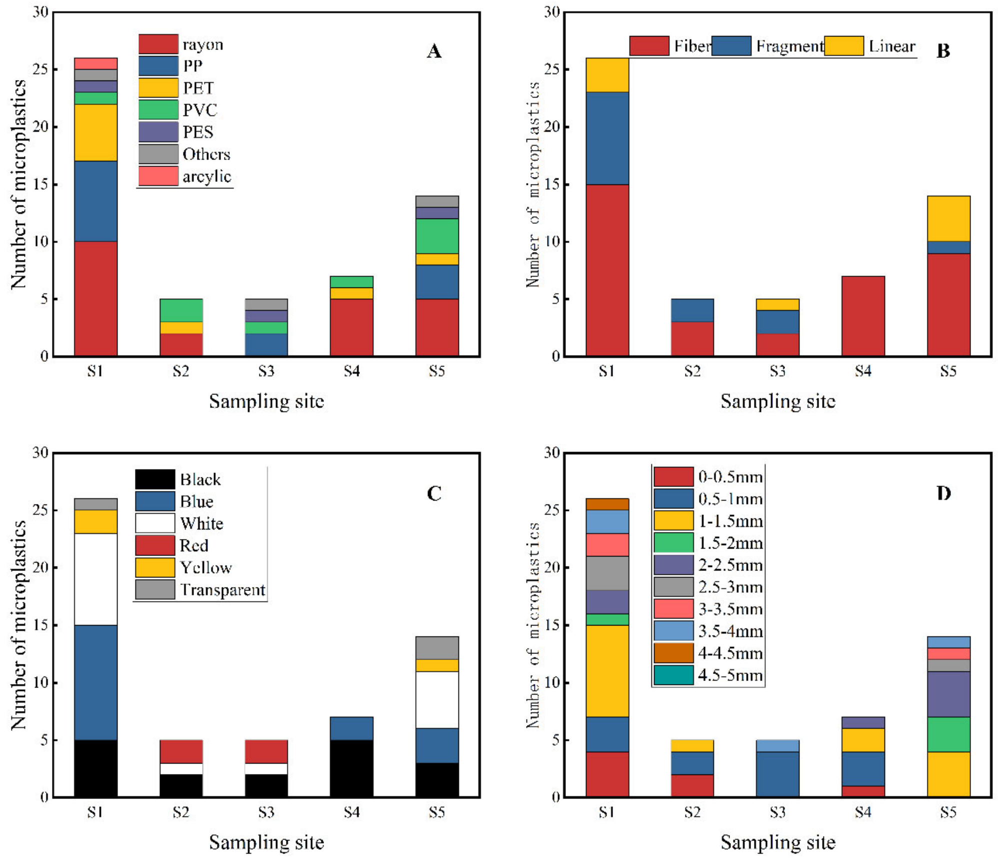

Figure S8. Characterization of microplastic contamination in soil, composition and number of microplastic materials at each sampling point (A), composition and number of microplastic shapes at each sampling point (B), composition and number of microplastic colors at each sampling point (C), composition and number of microplastic particle sizes at each sampling point (D)

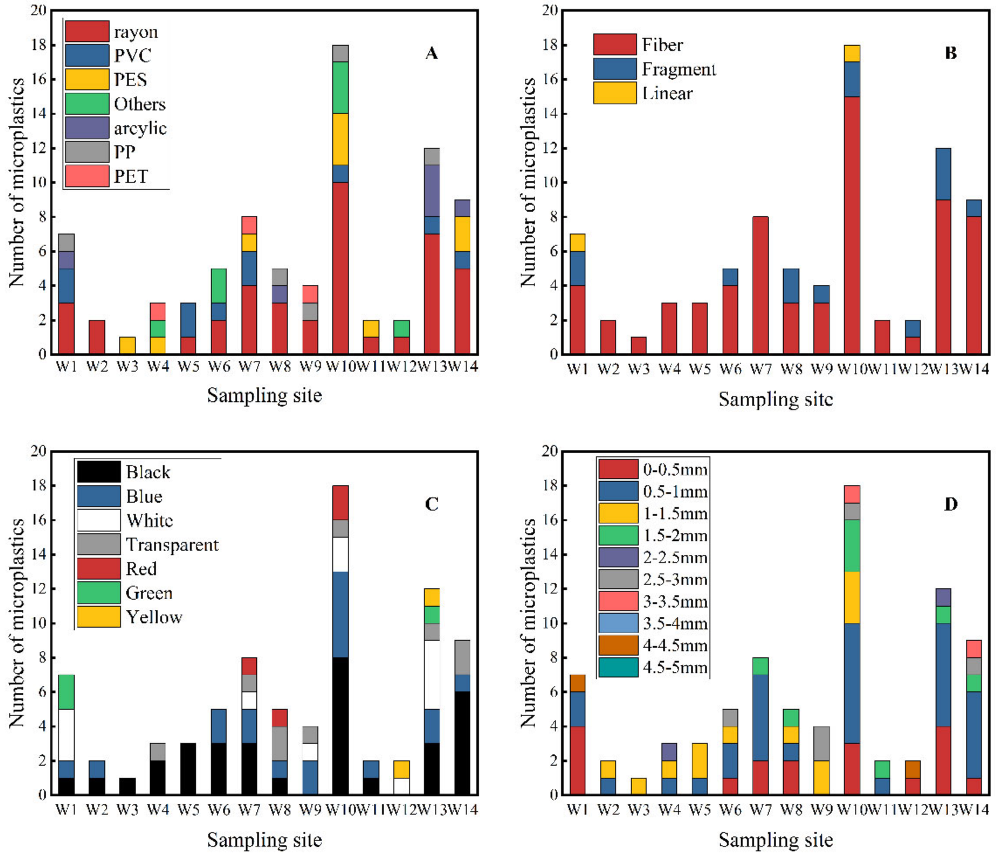

Figure S9. Characteristics of microplastic pollution in seawater, composition and number of microplastic materials at each sampling point (A), composition and number of microplastic shapes at each sampling point (B), composition and number of microplastic colors at each sampling point (C), and composition and number of microplastic particle sizes at each sampling point (D)

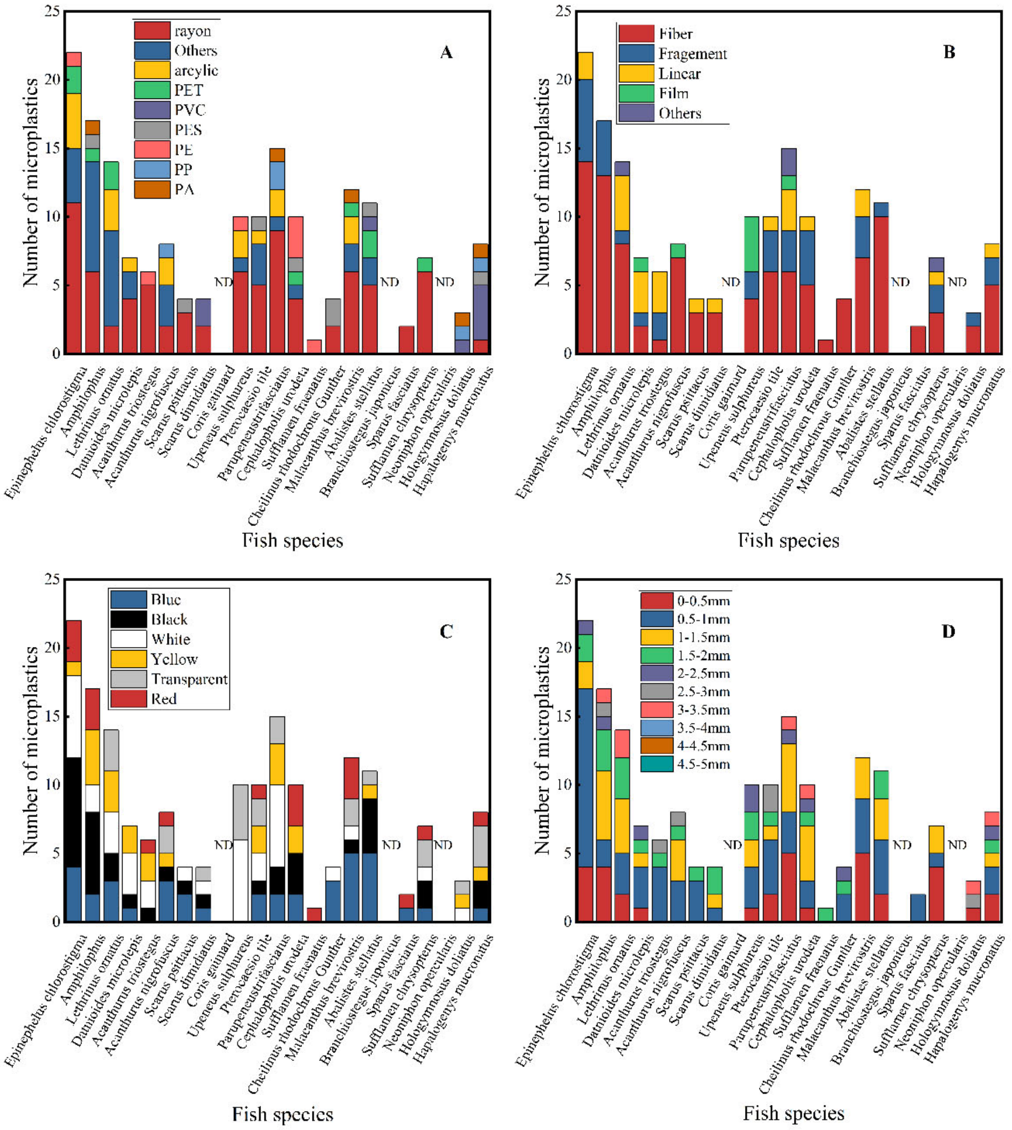

Figure S10.Characterization of microplastic contamination in fish samples, microplastic material composition and number of microplastics per species (A), microplastic shape composition and number of microplastics per species (B), microplastic color composition and number of microplastics per species (C), microplastic particle size composition and number of microplastics per species (D)

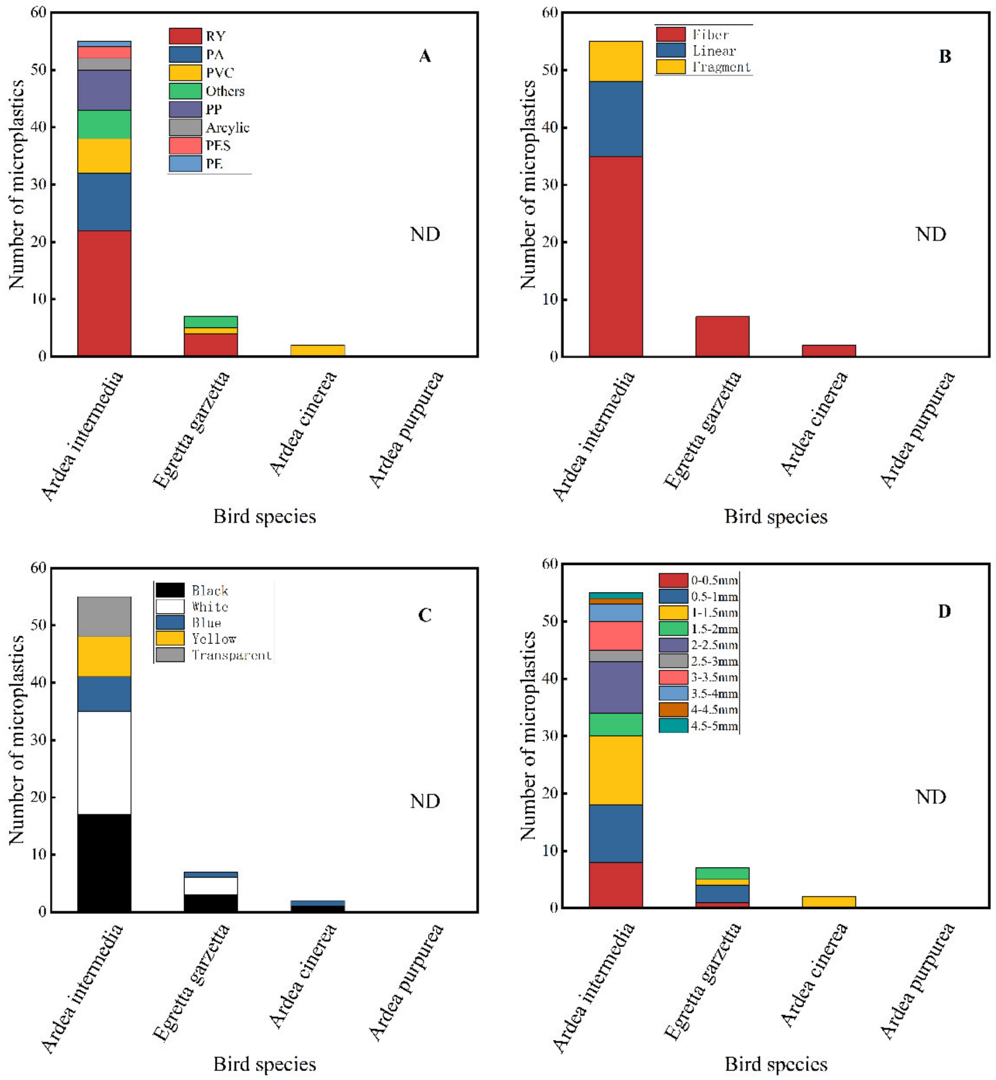

Figure S11. Characterization of microplastic contamination in bird samples, microplastic material composition and number of microplastics per species (A), microplastic shape composition and number of microplastics per species (B), microplastic color composition and number of microplastics per species (C), microplastic particle size composition and number of microplastics per species (D)

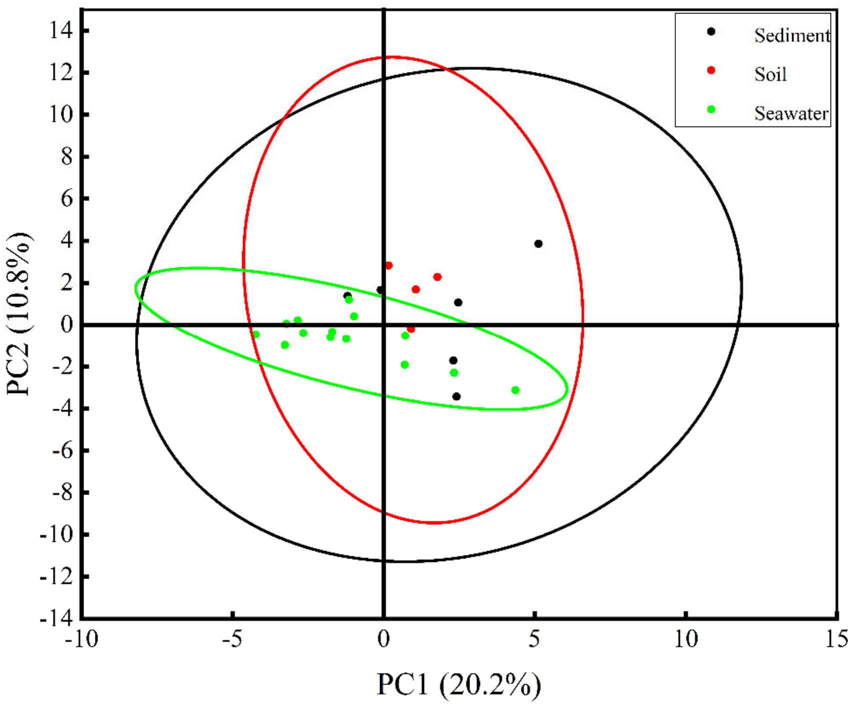

Figure S12. PCA characterization of microplastic contamination detected in different environmental media

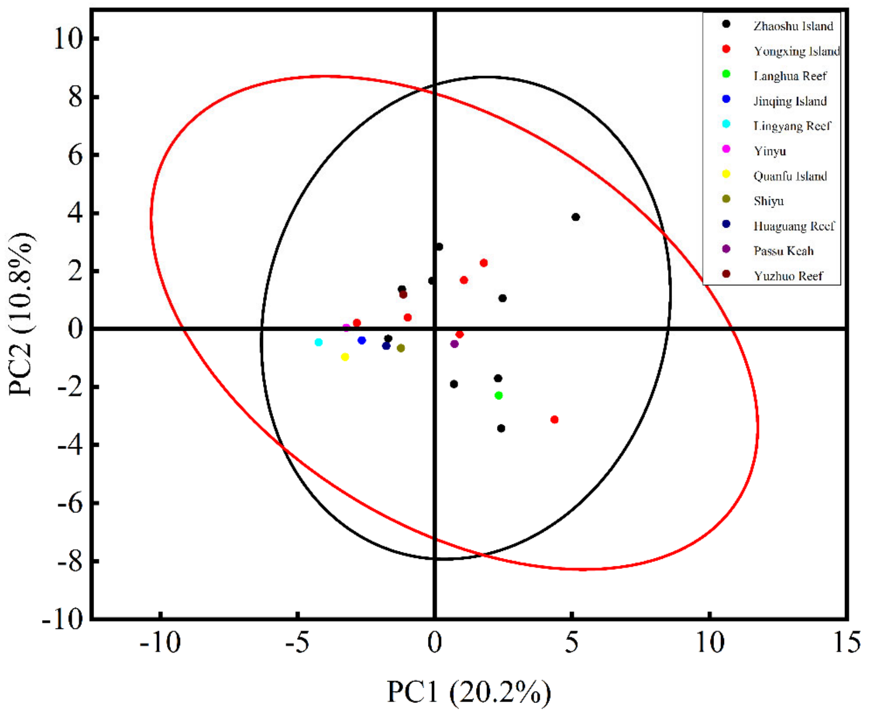

Figure S13. PCA analysis of microplastic contamination characteristics detected in different island environmental media

Table S5. Classification of Microplastic Pollution Load Index Levels

PLI	<10	10-20	20-30	>30
Risk Level	Ⅰ（low）	Ⅱ（medium）	Ⅲ（high）	Ⅳ（very high）

Table S6. Microplastics Risk Index Rating Scale

PHI	<10	10 - 100	100 - 1000	> 1000
Risk Level	Ⅰ（low）	Ⅱ（medium）	Ⅲ（high）	Ⅳ（very high）

Table S7. Microplastic hazard score values

Type	PP	PE	PET	PA	PVC	PS	Arcylic
Rating Value	1	11	4	47	5001	30	1021
Risk Level	Ⅰ	Ⅱ	Ⅰ	Ⅱ	Ⅳ	Ⅱ	Ⅳ

Table S8. Classification of potential ecological risk indices for microplastics

PERI	< 150	150 - 300	300 - 600	600 - 1200	> 1200
Risk Level	No risk	Low risk	Medium risk	High risk	Very high risk
